# Longitudinal [18F]FB-IL-2 PET Imaging to Assess the Immunopathogenicity of O'nyong-nyong Virus Infection

**DOI:** 10.3389/fimmu.2020.00894

**Published:** 2020-05-12

**Authors:** Yi-Hao Chan, Teck-Hui Teo, Anthony Torres-Ruesta, Siddesh V. Hartimath, Rhonda Sin-Ling Chee, Shivashankar Khanapur, Fui Fong Yong, Boominathan Ramasamy, Peter Cheng, Ravisankar Rajarethinam, Edward G. Robins, Julian L. Goggi, Fok-Moon Lum, Guillaume Carissimo, Laurent Rénia, Lisa F. P. Ng

**Affiliations:** ^1^Singapore Immunology Network, Agency for Science, Technology and Research (A^*^STAR), Immunos, Biopolis, Singapore, Singapore; ^2^National University of Singapore Graduate School for Integrative Sciences and Engineering, National University of Singapore, Singapore, Singapore; ^3^Department of Biochemistry, Yong Loo Lin School of Medicine, National University of Singapore, Singapore, Singapore; ^4^Singapore Bioimaging Consortium, A^*^STAR, Helios, Biopolis, Singapore, Singapore; ^5^Advanced Molecular Pathology Laboratory, Institute of Molecular and Cell Biology, A^*^STAR, Proteos, Biopolis, Singapore, Singapore; ^6^Clinical Imaging Research Centre, Yong Loo Lin School of Medicine, National University of Singapore, Singapore, Singapore; ^7^Institute of Infection and Global Health, University of Liverpool, Liverpool, United Kingdom

**Keywords:** O'nyong-nyong virus, alphavirus, immunopathogenesis, CD4+ T cells, drug repositioning, PET imaging

## Abstract

O'nyong-nyong virus (ONNV) is an arthritogenic alphavirus that caused two large epidemics in 1959 and 1996, affecting millions of people in Africa. More recently, sero-surveillance of healthy blood donors conducted in 2019 revealed high rates of unreported ONNV infection in Uganda. Due to similar clinical symptoms with other endemic mosquito-borne pathogens in the region, including chikungunya virus, dengue virus and malaria, ONNV infections are often un- or misdiagnosed. Elucidating the immunopathogenic factors of this re-emerging arbovirus is critical with the expanding geographic distribution of competent vectors. This study reports the establishment of an immune competent C57BL6/J mouse model to mechanistically characterize ONNV infection and assess potential treatment efficacy. This mouse model successfully recapitulated arthralgia and viremia profiles seen in ONNV patients. Furthermore, longitudinal *in-vivo* PET imaging with [^18^F]FB-IL-2 (CD25+CD4+ binding probe) and histopathological assessment in this model demonstrated the pathogenic role of CD4+ T cells in driving joint pathology. Concordantly, *in vivo* CD4+ T cell depletion, or suppression with fingolimod, an FDA-approved immunomodulating drug, abrogated CD4+ T cell-mediated disease. This study demonstrates the importance of this immune competent ONNV model for future studies on factors influencing disease pathogenesis, which could shape the discovery of novel therapeutic strategies for arthritogenic alphaviruses.

## Introduction

O'nyong-nyong virus (ONNV) is a mosquito-borne arthritogenic alphavirus. ONNV-infected patients display acute clinical symptoms of low-grade fever, self-limiting polyarthralgia, and other disease manifestations like lymphadenopathy, generalized maculopapular skin rash, and rare occurrences of bleeding gums and nosebleeds (1, 2). There are also cases of prolonged joint pain during the convalescence phase, although no further complications are reported (1). ONNV cases are often underestimated and misdiagnosed for other endemic infectious diseases with more pronounced, overlapping symptoms, such as chikungunya virus (CHIKV), dengue virus (DENV) or malaria.

ONNV has been the cause of few, but major epidemics confined to Africa. It was first discovered in 1959, during a large outbreak in northern Uganda that affected more that 2 million people (2, 3). After a 35-year hiatus, ONNV re-emerged with two outbreaks in 1996 and 2003 in southern-central Uganda and Ivory Coast, respectively, affecting thousands of people (1, 4–6). Antibody-based serological assays highlighted the nearly complete cross-reactivity between CHIKV and ONNV (6–8), hindering proper detection and diagnosis. Concordantly, recent serological studies revealed high proportions of previously unreported alphavirus-seropositive coastal Kenya and Uganda populations, particularly to ONNV and closely-related CHIKV (4, 9, 10). The absence of epidemiological data on transmission levels in Africa and other regions highlight ONNV as an arbovirus with high potential of global spread. Currently, the components of host immunity mediating ONNV disease are poorly understood. Establishing a mouse model which recapitulates ONNV clinical symptoms is thus an essential step for the characterization of ONNV pathogenesis and the development of anti-viral therapeutic strategies.

This study reports the development of an immune-competent C57BL6/J mouse model, which upon ONNV infection, presents a self-limiting disease with peak viremia at 3 days post-infection (dpi) and peak joint inflammation at 6 dpi. ONNV-induced joint pathology was further evaluated with tracer assay and histopathological analyses. In addition, immunophenotyping and flow cytometry analyses of immune subsets in the infected joints revealed the pathogenic roles of CD4+ T cells in ONNV infection. Subsequently, longitudinal [^18^F]FB-IL2 PET imaging was performed to evaluate the effectiveness of FDA-approved immune-modulatory drug, fingolimod, as a therapeutic strategy to inhibit CD4+ T cell infiltration and abrogate joint swelling. The development of this mouse model will prove essential in future research to characterize the immune-pathogenesis of ONNV and the *in vivo* assessment of potential therapeutics.

## Materials and Methods

### Virus

The WT ONNV isolate (IMTSSA/5163) was isolated from a patient in Chad in 2004 (kindly provided by Marc Grandadam from Unité de Virologie Tropicale, IMTSSA, Marseille, France) (11). Virus isolation was attempted by incubation of patient peripheral blood mononuclear cells collected on day of illness onset with C6/36 (Aedes albopictus) (ATCC CRL-1660) and Vero E6 (ATCC CRL-1586) monolayers. Supernatants were collected 5 days later and passaged once more in fresh cell cultures to produce the virus stock. Virus stock was tested by indirect immunofluorescene assay (IFA) and qualitative reverse transcriptase real time polymerase chain reaction (qRT-PCR) to be negative for other alphaviruses. Virus stock was further propagated in C6/36 cells and purified on a 20% (w/v) sucrose-cushion ultracentrifugation to produce the ONNV infection stock used in mice, as previously described (12). Infection stock titers were determined by standard plaque assay using Vero E6 cells, with a viral RNA/PFU ratio of 1440.

Firefly luciferase (Fluc)-tagged ONNV infectious clone (ONNV-Fluc), with the Fluc gene inserted between the two open reading frames was produced using a similar methodology as previously described (13). Expression of Fluc gene is regulated by a second sub-genomic promoter. ONNV-Fluc was propagated in C6/36 and virus titers were determined by standard plaque assay using Vero E6 cells.

### Mice

Three-week-old and 6-week-old gender-matched wild-type (WT) C57BL/6J mice were bred and kept in specific pathogen-free conditions in the Biological Resource Center (BRC) of Agency for Science, Technology, and Research, Singapore (A^*^STAR). Experimental procedures involving mice were approved by the Institutional Animal Care and Use Committee (IACUC #181353) of A^*^STAR, and in compliance to the guidelines of the Agri-Food and Veterinary Authority (AVA) and the National Advisory Committee for Laboratory Animal Research of Singapore (NACLAR).

### Virus Infection and Disease Evaluation

Mice were inoculated subcutaneously with 10^6^ plaque forming units (PFU) of wild-type ONNV in 30 μl of Dulbecco's Phosphate-Buffered Saline (PBS) at the ventral side of the right hind footpad. Viremia was monitored daily for 2-weeks. Height (thickness) and breadth measurements were done for the metatarsal region of the ONNV infected joint daily for 2-weeks, and quantified as (Height × breadth). The disease score was then expressed as the relative fold change in foot size compared with the same foot before infection (0 dpi) using the following formula: [(*x* –0 dpi)/0 dpi], where *x* is the quantified footpad measurement for each respective day.

### Viral RNA Extraction and Viral Copies Quantification

Ten microliters of blood were obtained from the tail vein, and re-suspended in 120 μl of PBS supplemented with 10 μl of citrate-phosphate-dextrose solution (Sigma-Aldrich). The viral RNA in the blood samples were purified by QIAamp Viral RNA kit (Qiagen), according to the manufacturer's protocol. Viral RNA is eluted in 60 μl of elution buffer. Viral load in 1 μl of the elution buffer was subsequently quantified by qRT-PCR using QuantiTect Probe RT PCR kit (Qiagen). For ONNV viral genome quantification, the following primers were designed to amplify negative nsP1 viral RNA: forward primer (AATTACGCGAGAAAACTTGCG), reverse primer (TTTTTCCAGAGATGTTTTTATCTGT) and TaqMan Probe (CCGCTGGAAAGGT), as described previously (14). The cycling conditions used are as follows: 1) 50°C for 30 min; 2) 95°C for 15 min; 3) 45 cycles of 94°C for 15 s and 55°C for 1 min. Data collection occurred during the 55°C extension step (15).

### Mouse Joint Cell Isolation

ONNV-infected mice were euthanized at 6 dpi. Joints were harvested at the ankles, deskinned, and placed in 4 ml of digestion medium containing dispase I (2 U/ml; Invitrogen), collagenase IV (20 μg/ml; Sigma-Aldrich), and DNase I mix (50 μg/ml; Roche Applied Science) in RPMI supplemented with 10% fetal bovine serum (FBS). Joints were incubated in digestion medium for 4 h at 37°C, 5% CO_2_ on a shaker. Digested tissues were then placed and grounded in 40 μm cell strainer with 1 ml syringe plunger. Red blood cells were lysed using ammonium chloride solution. Samples were then washed and resuspended in 2 ml complete RPMI medium, and overlaid onto 1 ml 35% v/v Percoll/ RPMI medium (Sigma-Aldrich) for gradient centrifugation at 2,400 rpm for 20 min. Cells were washed and resuspended in complete RPMI before counting with hemocytometer (16).

### Phenotyping of Leukocytes by Flow Cytometry

Isolated cells were incubated with blocking buffer containing 1% rat and mouse serum prior to staining. Cells were stained with BUV395-conjugated rat IgG_2b_ anti-mouse CD45 (clone 30-F11; BD Biosciences), Pacific Blue-conjugated rat IgG_2a_ anti-mouse CD4 (clone RM4-5; BioLegend), PE-CF594-conjugated rat IgG_2a_ anti-mouse CD8 (clone 53-6.7; BD Biosciences), PE-Cy7-conjugated rat IgG_2b_ anti-mouse CD3 (clone 17A2; BioLegend), APC-Cy7-conjugated rat IgG_2c_ anti-mouse Ly6C (clone HK1.4; BioLegend), Alexa Flour 700-conjugated rat IgG_2b_ anti-mouse MHC-II (clone M5/114.15.2; BioLegend), PerCP-Cy5.5-conjugated rat IgG1 anti-mouse LFA-1(clone H155-78; BioLegend), BV650-conjugated rat IgG_2b_ anti-mouse CD11b (clone M1/70; BioLegend), BV605-conjugated Armenian hamster IgG anti-mouse CD11c (clone N418; BioLegend), PE-CF594-conjugated rat IgG_2a_ anti-mouse Ly6G (clone 1A8; BD Biosciences), eFluor450-conjugated rat IgG_2a_ anti-mouse B220 (clone RA3-6B2; eBio- science), APC-conjugated rat IgG_1_ anti-mouse CD64 (clone X54-5/7.1; BioLegend), Biotin-conjugated mouse IgG_2a_ anti-mouse NK1.1 (clone PK136; eBioscience), and BUV737 streptavidin (BD Biosciences) for 20 min at room temperature. Cells were then washed and fixed with 50 μl of intracellular (IC) fixation buffer (eBioscience) for 5 min followed by acquisition with LSR II (5 laser) flow cytometer (BD Biosciences) with FACSDiva software and analyzed with FlowJo software (version 10.4.2).

### High Dimensional Analysis of Flow Cytometry Data

Live CD45+ singlets events were pre-gated and then randomly down-sampled to a fixed number (*n* = 5,000) for each sample. Logical transformation was performed using autoLgcl of the Bioconductor package cytofkit2. The dimensionality algorithm UMAP was then run using 1,000 max iterations, 30 nearest neighbors and other default parameters (17–19). For clustering, PhenoGraph (R package Rphenograph v0.99.1) was used with default parameters (*k* = 30).

### [^18^F]FB-IL-2 Radiochemistry and PET-CT Imaging

*N*-(4-[^18^F]fluorobenzoyl)-interleukin-2 ([^18^F]FB-IL2) was prepared in a two-step reaction using a previously reported method with minor modifications (20). First, *N*-succinimidyl 4-[^18^F]fluorobenzoate ([^18^F]SFB) synthon was prepared by reacting the 4- (ethoxycarbonyl)- *N,N,N*- trimethylbenzenaminium trifluoromethanesulfonate (precursor for SFB synthesis) with azeotropically dried [^18^F]fluoride. The HPLC purified [^18^F]SFB was trapped into an Oasis HLB plus light cartridge and eluted with diethyl ether. The solvent was removed under nitrogen flow and [^18^F]SFB was reconstituted in 100 μL DMSO. The reconstituted [^18^F]SFB was added dropwise into a mixture of 100 μL aqueous IL-2 solution (Proleukin® 2 mg/ml) and 100 μL sodium borate buffer (1 M, pH 8.5) and incubated at 50°C for 10 min. After the reaction, the crude mixture was purified by size-exclusion chromatography using a PD-10 column, which was previously conditioned with 30 mL elution buffer (0.05% SDS in PBS). Pure labeled [^18^F]FB-IL-2 protein was eluted in 4–5 fractions of 0.5 mL each and pooled. The radiochemical purity of [^18^F]FB-IL-2 was determined using analytical radio-HPLC and the purity was >97% with good specific activity (114 ± 94 Gabs/μmol).

PET-CT scanning was performed on 0, 4, and 6 days after ONNV infection using a Siemens Inveon PET-CT. Briefly, animals were sedated using 1.5% inhaled isoflurane and injected with [^18^F]FB-IL-2 (~10 MBq per animal) via the lateral tail vein. A 10 min static PET acquisition was performed 60 min post tracer-injection. CT scans were performed for anatomical co-registration and attenuation correction. During the scanning, animals were monitored for body temperature and respiration rate using a Biovet physiological monitoring system. Post image-analysis of reconstructed calibrated images were performed with FIJI and Amide software (version 10.3 Sourceforge). Uptake of radioactivity in the paw region was determined by the placement of a region of interest (ROI) over the paw delineated by CT images. The [^18^F]FB-IL-2 uptake was expressed as percentage of injected dose per gram of tissue (%ID/g).

### Depletion of CD4+ T Cells

CD4+ T cell depletion was performed as previously described (21). Briefly, mice were injected intraperitoneally with 500 μg of CD4-depletion antibody (InVivoPlus rat anti-mouse CD4, Bio X Cell, #BP0003-1) or rat IgG control (Sigma-Aldrich) on −1 and +4 dpi. CD4+ T cell depletion was confirmed by flow cytometry with antibodies that stain for CD4+ T cells [BUV395-conjugated rat IgG_2b_ anti-mouse CD45 (clone 30-F11; BD Biosciences), Pacific Blue-conjugated rat IgG_2a_ anti-mouse CD4 (clone RM4-5; BioLegend), PE-Cy7-conjugated rat IgG_2b_ anti-mouse CD3 (clone 17A2; BioLegend)] before ONNV inoculation (0 dpi). Mice with incomplete depletion of CD4+ T cells were administered with another dose of CD4-depletion antibody on the same day.

### Fingolimod Treatment

Fingolimod (FTY720) (Caymen Chemical) was reconstituted in DMSO and subsequently diluted to working concentration (0.8%) with PBS in sterile conditions. As a therapeutic treatment, mice were administered intraperitoneally with 20 μg (~1 mg/kg) of the drug daily from 2 to 6 dpi.

### Quantification of Joint Vascular Leakage by Tracer Assay

Tracer 653 Assay (Molecular Targeting Technologies Inc.) was performed as described (22). Briefly, 100 μl of tracer dye was injected intravenously and allowed to circulate passively in each mouse for 1 h. Tracer dye was then quantified with *in vivo* imaging system (IVIS) (PerkinElmer). Vascular leakage in regions of interest were quantified using Living Image 3.0, and average radiance in selected areas [infected joint, Log_10_(p/s/cm^2^/sr)] were determined.

### Histology

Mice were euthanized on 6 dpi, and perfused with PBS, followed by 10% neutral buffered formalin (NBF). ONNV-infected joints were then harvested at the ankles, and fixed in 10% NBF for 24 h at room temperature. The joints were then decalcified in Osteosoft (Merck, Germany), and trimmed into three portions at 5 mm intervals. Sectioned tissues were then routinely processed, sectioned into 5 μm thickness, and stained with Hematoxylin and Eosin (H&E). Tissues were then viewed under an Olympus BX53 upright microscope (Olympus Life Science, Japan) and images were captured with an Olympus DP71 digital color camera with Olympus DP controller and DP manager software. Histo-pathological assessments were performed in a blind fashion by histo-pathologist, using a scoring method in each individual animal based on the presence of edema, inflammation, muscle necrosis, tendonitis and synovitis, if any. Severity of the lesions were assigned to the following scale: 0—no finding; 1—minimal; 2—mild; 3—moderate; 4—marked; 5—severe; as previously described (23).

### Statistical Analysis

Data are presented as mean ± SD unless specified otherwise. Statistical differences between infected and control groups were analyzed using unpaired non-parametric Mann-Whitney *U*-statistical test unless specified otherwise in the specific results section and figure legends. Median centering was applied to the data to reduce variation between experiments when necessary. Statistics were performed with GraphPad Prism 7.0a (GraphPad Software). *P*-values considered statistically significant are represented with ^*^P < 0.05, ^**^P < 0.01, and ^***^P < 0.001.

## Results

### O'nyong-nyong Virus (ONNV) Infects Wild-Type C57BL/6J Mice

**Figure 1:**
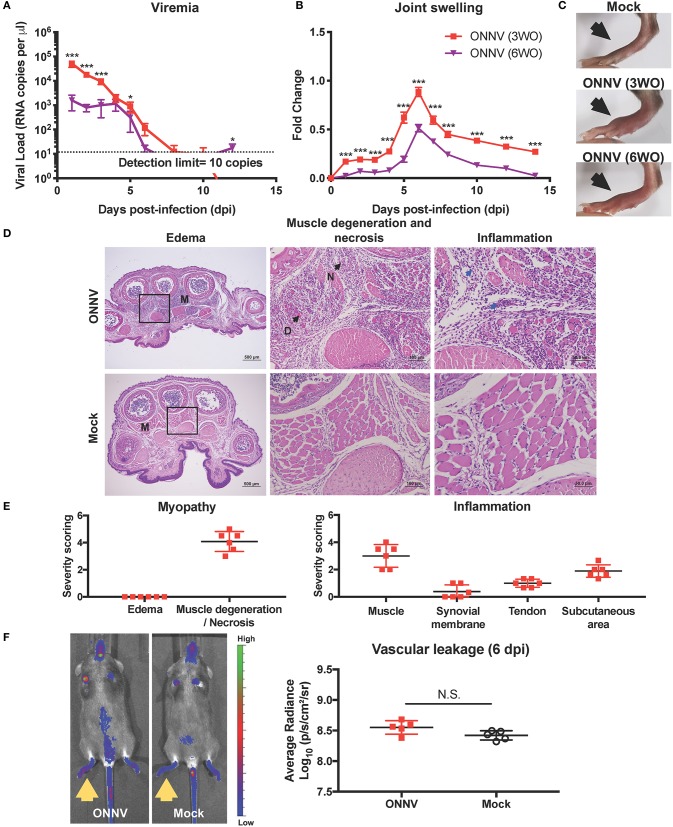
ONNV infection in wild-type C57BL/6 mice resulted in virus replication and joint pathology. **(A)** Viremia and **(B)** joint swelling of ONNV-infected mice aged 3-weeks-old (3WO, *n* = 13) and 6WO (*n* = 14) were monitored over 2-weeks. Data are representative of 2 independent experiments and presented as mean ± SEM. Statistical analyses were performed using two-tailed Mann Whitney *U* test (^*^P < 0.05; ^***^P < 0.001). **(C)** Representative joint images at 6 dpi **(D)** Representative hematoxylin and eosin (H&E) images of inflamed joint footpad on 6 dpi. The muscle area (M) shows presence of muscle degeneration (D) and necrosis (N) upon ONNV infection. Blue arrows indicate infiltration of immune cells upon ONNV infection. Magnified area is indicated by the box. **(E)** Severity scores were assigned for myopathy, including edema and muscle degeneration and necrosis, and inflammation at the muscle, synovial membrane, tendon and subcutaneous region (*n* = 6). None of these pathological distortions were observed in non-infected mice. **(F)** Representative fluorescence images of the tracer assay, with inflamed joints indicated by yellow arrows, and quantification of joint vascular leakage into the joints of ONNV-infected and PBS control groups (*n* = 5 per group). Data is presented as mean ± SD. No statistical significance was observed between the two groups with two-tailed Mann Whitney *U*-test.

To optimize an *in vivo* pre-clinical model for ONNV, one million infectious ONNV particles were subcutaneously inoculated into the right-hind joints of 3-week-old (WO) or 6WO C57BL/6J mice, and the progression of viremia and joint inflammation were monitored for 14 days. Both 3WO and 6WO mice developed self-limiting viremia and arthralgia, which recapitulated the clinical symptoms observed in patients (1, 2). Notably, 3-week-old mice suffered from significantly higher levels of viral load in the peripheral blood compared to 6WO mice. At 6 days post-infection (dpi), viremia from both groups start to drop below detection limit of 10 genome copies per μl (Figure 1A). Interestingly, while viremia in 6WO mice plateaued from 1 to 4 dpi, the levels were highly variable within the group, with 20 to 40% of mice having viremia below detection limit during this period suggesting that 3WO mice are a more consistent model for investigating *in vivo* viral resolution (Figure 1A). To confirm that viremia in the early acute phase was representative of viral replication instead of inoculum decay, we constructed a ONNV reporter infectious clone carrying a Firefly luciferase gene expressed only during replication (24). This confirms that viral replication can be observed as early as 6 h post-infection (hpi), and peaks at 12 hpi in 3WO mice (Supplementary Figure 1). The luciferase signal can be detected in the ONNV-infected joints up to 40 dpi, which is similar to CHIKV infection (16).

ONNV-infected joints developed a mild, yet visible joint swelling around the metatarsal region that peaked on 6 dpi (Figures 1B,C). Importantly, peak joint swelling was significantly more pronounced for the 3WO group (Figures 1B,C). Together, the results show that the 3WO C57BL/6J mouse is a good immune-competent pre-clinical model for ONNV as it recapitulates most of the clinical symptoms.

### ONNV Infection Induces Muscle Damage and Infiltration of Inflammatory Cells

Histopathological analyzes in the ONNV-infected joint, performed on the peak of joint swelling (6 dpi), revealed the presence of marked muscle degeneration and necrosis, mild synovitis at the synovium, and tenosynovitis around the tendon capsule (Figures 1D,E). There were also detectable amounts of immune cells in the muscle, synovial membrane, tendon, and subcutaneous area of the infected joint (Figures 1D,E). Interestingly, no edema was detectable in the ONNV infected joints (Figures 1D,E). This observation suggests that the joint swelling was not due to vascular leakage but contributed by the tissue damage in the joints.

To validate the absence of vascular leakage in ONNV-infected joints, tracer assay and *in vivo* imaging were performed. At the peak of joint swelling, a marginal and non-significant increase of tracer dye could be detected in ONNV-infected joints as compared to mock-infected controls (Figure 1F). Together, these results show that ONNV infection does not cause a detectable edema and vascular leakage in the joints.

### High-Dimensional Analysis of Immune Infiltrates Highlights Involvement of CD4+ T Cells

**Figure 2:**
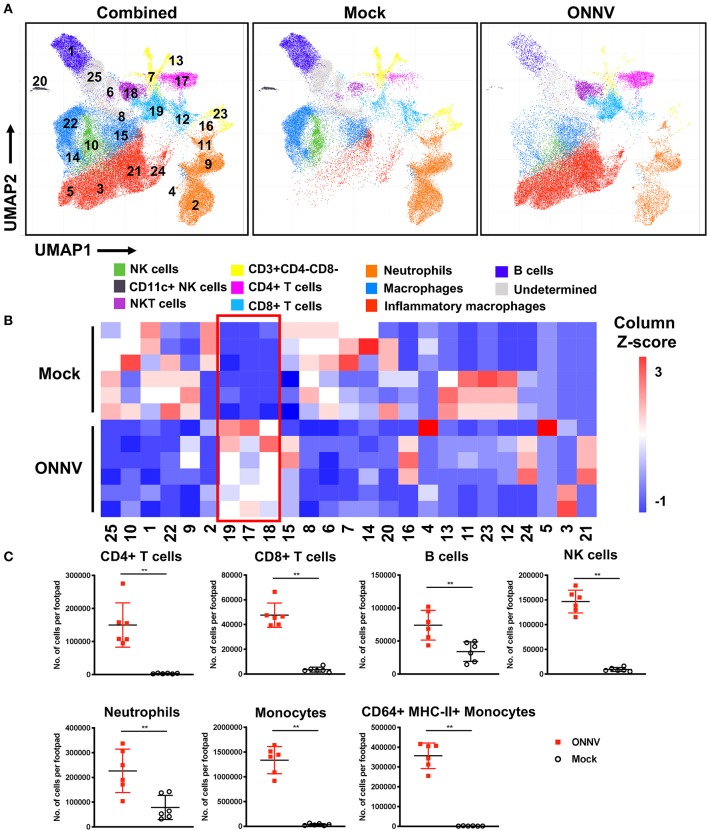
High-dimensional analysis of flow cytometry data reveals differences in joint immune profile upon ONNV infection. Joints from ONNV- and mock-infected 3-week-old WT C57BL/6 mice were harvested at 6 dpi. **(A)** Visualization of clusters in UMAP plots for combined data or individual groups (*n* = 6 per group). UMAP plots shown consists of 30,000 cells each and are representative of concatenated samples within each group. Clusters are grouped in proximity based on lineage marker similarities and are not representative of conventional cell types. Populations corresponding to NK cells (green), CD11c+NK cells (black), NKT cells (purple), immature T cells (yellow), CD4+ T cells (magenta), CD8+ T cells (light blue), neutrophils (orange), macrophages (blue), inflammatory macrophages (red), B cells (dark blue) and three undermined clusters (gray) are shown. **(B)** Cell percentage heatmap of PhenoGraph clusters (1 to 25) across ONNV- and mock-infected individual mice (*n* = 6 per group). Red box indicates the upregulated clusters during 6 dpi. **(C)** Leukocyte subsets were identified by antibody staining based on lineage surface marker expression. Leukocyte subsets in ONNV-infected joints at 6 dpi were quantified, including CD4+ T cells, CD8+ T cells, B cells, NK cells, neutrophils, monocytes and CD64+ MHC-II+ monocytes. Data is representative of two independent experiments and presented in mean ± SD. Statistical analyses were performed using two-tailed Mann Whitney *U*-test (^**^P < 0.01).

The development of joint swelling and pathology during ONNV infection could be driven by the immune-pathogenesis of infiltrating leukocytes into the joints. To characterize the immune-cell profiles at the peak of inflammation, ONNV-infected joints were harvested at 6 dpi, and cells were subsequently isolated and processed for immune-phenotyping via flow cytometry. Uniform Manifold Approximation and Projection, UMAP (a dimension reduction technique used for data visualization similar to t-SNE) (17, 19) and PhenoGraph (25) were performed on this high-dimensional cytometry dataset to obtain 25 clusters of cells with homogenous marker expression profiles (Figure 2A). These clusters were then identified based on the collective expression levels of lineage markers (Supplementary Figure 2A). The UMAP biplots were normalized to 5,000 cells per joint (*n* = 6 per group) and represent the proportion, but not the quantity of cells in the joint. As observed from the biplots, ONNV infection resulted in the activation of macrophages (clusters 14, 15, 22) to CD64+ Ly6C+ MHCII+ inflammatory macrophages (clusters 3, 5, 21, 24) (Figure 2A). Interestingly, CD4+ T cells (cluster 17) and CD8+ T cells (cluster 12, 19) were highly upregulated during ONNV infection, in conjunction with the decrease of naïve CD3+ T cells (cluster 7) (Figures 2A,B).

To quantify the number of cells infiltrating into the joints upon ONNV infection, cells acquired from the flow cytometry were sorted into the major leukocyte subsets based on the surface expression of lineage markers (Figure 2C and Supplementary Figure 2B). Several immune cell subsets were present in the infected joints at 6 dpi, notably CD4+ T cells, CD8+ T cells, B cells, NK cells, neutrophils, monocytes and activated CD64+ MHC-II+ monocytes (Figure 2C).

### CD4+ T Cells Mediate Inflammation and Muscle Necrosis During Arthritogenic ONNV Infection

**Figure 3:**
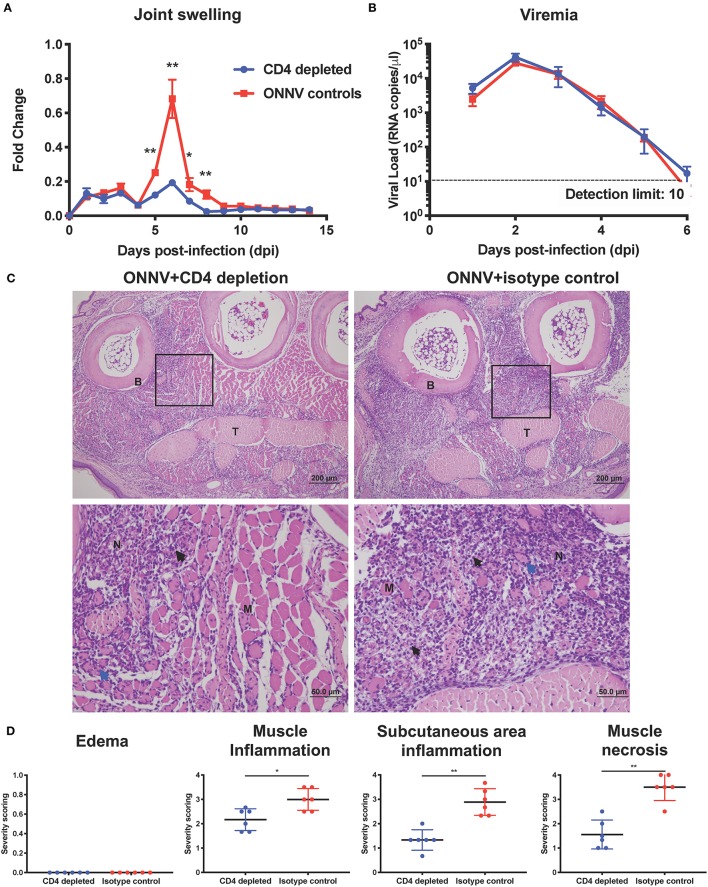
Arthritogenic CD4+ T cells mediate joint pathology. **(A)** Joint swelling and **(B)** viremia of ONNV+CD4 depletion (*n* = 6) and ONNV + isotype control (*n* = 5) groups were monitored over 2-weeks. Statistical analyses were performed using two-tailed Mann Whitney *U* test (^*^P < 0.05; ^**^P < 0.01). Data points of viremia beyond 6 dpi were below detection limit. Data is representative of 2 independent experiments and presented as mean ± SEM. **(C)** Representative photo micrographs of inflamed joint footpad at 6 dpi. B, bone; T, tendon; M, skeletal muscle; black arrow, infiltration of inflammatory cells; blue arrow, degeneration of muscle; N, necrosis of muscle. Magnified area is indicated by the box. **(D)** Histopathological scoring of edema, inflammation in different regions of the joint footpad and muscle pathology of ONNV-infected mice (*n* = 6) on 6 dpi was done in a blinded fashion. Scoring was done on three sections from each joint footpad, and data are expressed as mean ± SD. Statistical analyses were performed using two-tailed Mann Whitney *U*-test (^*^P < 0.05; ^**^P < 0.01).

**Figure 4:**
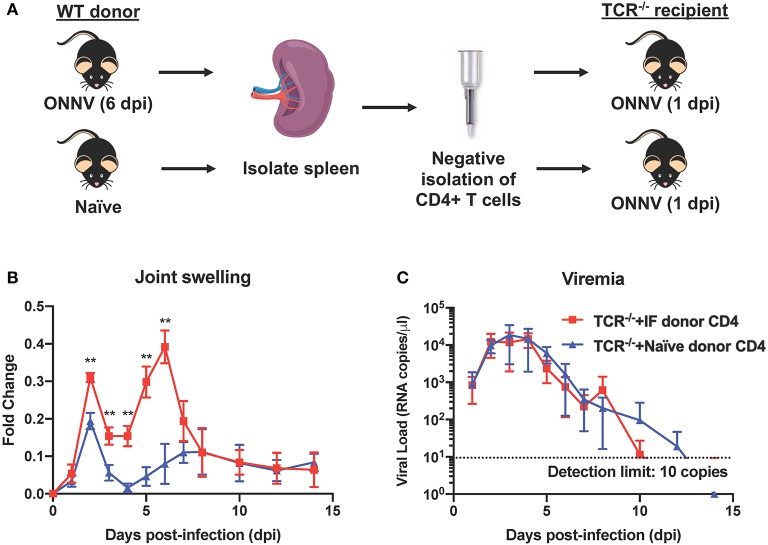
Adoptive transfer of CD4+ T cells into ONNV-infected TCR^−/−^ mice re-establishes virus-induced joint swelling. **(A)** CD4+ T cells were negatively isolated from spleens of ONNV-infected or mock-infected WT C57BL/6 donors. Isolated CD4+ T cells were then transferred into respective groups of recipient TCR^−/−^ mice (1 spleen to 1 recipient). **(B)** Joint swelling and **(C)** viremia of ONNV-infected recipient TCR^−/−^ mice (*n* = 5 per group) were monitored over 2-weeks. Data are presented as mean ± SD. Statistical analyses were performed using two-tailed Mann Whitney *U*-test (^**^P < 0.01).

Since CD4+ T cells were shown possess pathogenic roles in multiple alphavirus infections (16, 21, 26–28), we next decided to elucidate their role during ONNV infection. Mice depleted of CD4+ T cells were infected with ONNV and subsequently monitored for disease pathogenesis. Absence of CD4+ T cells significantly abrogated the severity of peak swelling by ~0.5-fold at 6 dpi (*P* = 0.0043), with no effect on viremia (Figures 3A,B). In addition, histopathological analyses also revealed significantly less inflammation in the muscle and subcutaneous area, and lower levels of muscle necrosis (Figures 3C,D). Concordantly, adoptive transfer of CD4+ T cells re-established joint swelling in ONNV-infected TCR^−/−^ animals (Figures 4A–C). Notably, the introduction of CD4+ T cells had no effect on viremia clearance (Figure 4C). Collectively, these observations confirm that CD4+ T cells contribute to ONNV-induced joint pathology.

### Therapeutic Treatment With Fingolimod Alleviates ONNV-Induced Joint Inflammation by Suppressing Pathogenic CD4+ T Cell Infiltration into Infected Joints

**Figure 5:**
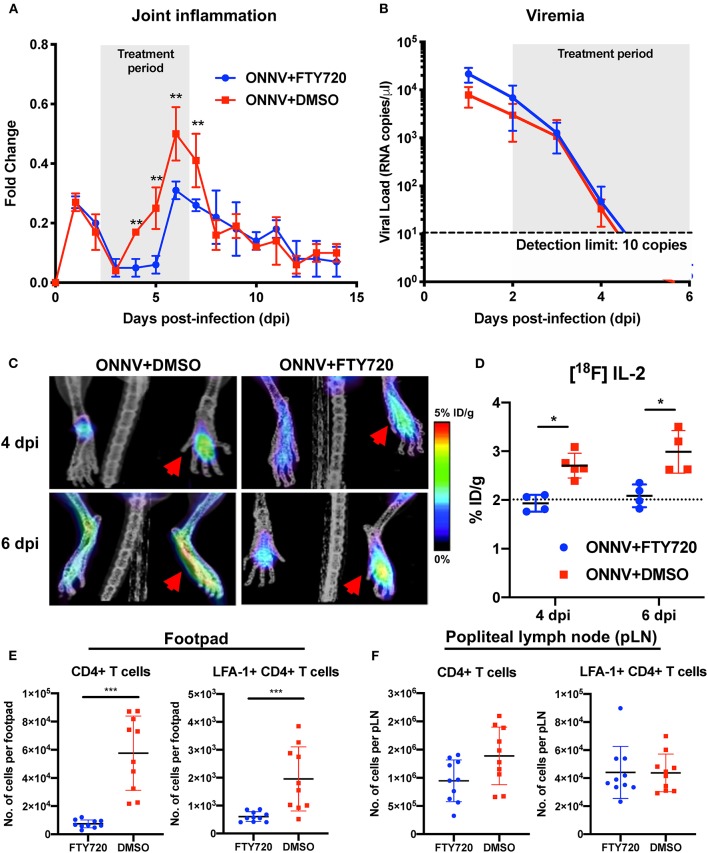
Therapeutic fingolimod (FTY720) treatments alleviate ONNV-induced joint pathology by inhibiting CD4+ T cells infiltration. **(A)** Joint inflammation and **(B)** viremia of ONNV+FTY720 treated groups and ONNV non-treated controls (*n* = 6 per group). Treated mice were intraperitoneally administered with FTY720 (20 μg) daily from 2 to 6 dpi, as denoted by shaded region. Viremia data beyond 5 dpi were below detection limit. Data are expressed as mean ± SD. Statistical analyses were performed using two-tailed Mann Whitney *U*-test (^**^P < 0.01). **(C)** Representative radiolabeled IL-2 uptake by CD25 of activated T cells in FTY720-treated and mock-treated ONNV infected mice. Red arrows indicated the paws infected with ONNV. **(D)** Percentages of injected [^18^F]FB-IL-2 dose per gram tissue were quantified in ONNV-infected mice treated with FTY720 or DMSO at 3 hpi, 4 and 6 dpi. Dotted line represents the radiolabeled IL-2 uptake baseline at 3 hpi. Data are expressed as mean ± SD. Statistical analyses were performed using two-tailed Mann-Whitney *U* test (^*^P < 0.05). **(E)** Activated CD4+ T cells were quantified in the joints and **(F)** popliteal lymph nodes (pLN) of FTY720-treated groups and non-treated controls (*n* = 10 per group). Data are expressed as mean ± SD and are representative of two independent experiments. Statistical analyses were performed using two-tailed Mann Whitney *U* test (^***^P < 0.001).

It was previously shown for a closely-related alphavirus, CHIKV, that inhibition of CD4+ T cell infiltration with an FDA-approved drug could reduce disease phenotype in the virus-infected joint (28). Therefore, we assessed if a similar treatment strategy would be beneficial against CD4+ T cell-induced pathology during ONNV infection. ONNV-infected mice were subjected to a post-infection treatment regimen of fingolimod (FTY720, 20 μg daily from 2 to 6 dpi). Treatment successfully abrogated joint swelling, with no effect on viremia (Figures 5A,B).

The immunomodulatory effects of FTY720 treatment on pathogenic CD4+ T cell infiltration into ONNV-infected joints were further investigated longitudinally with PET-CT imaging (Figure 5C and Supplementary Video 1). Notably, the amount of [^18^F]FB-IL-2 uptake in the joints of FTY720-treated mice were limited to basal levels (2% ID/g), indicating that treatment inhibited the infiltration of activated CD4+ T cells into the infection site (Figure 5D). To confirm this, flow cytometry phenotyping of the infiltrating cells was performed at 6 dpi on the infected joints and the draining popliteal lymph node (pLN). FTY720 treatment efficiently suppressed the ingress of CD4+ T cells and activated LFA-1+ CD4+ T cells into the virus-infected joints (Figure 5E). Interestingly, while levels of activated LFA-1+ CD4+ T cells were similar, there was a slightly lower amount of total CD4+ T cells in the pLN (Figure 5F), suggesting that FTY720 could also inhibit CD4+ T cell ingress or proliferation in the pLN.

## Discussion

The current existing *in vivo* models for the study of ONNV pathogenesis are typically immune-deficient models and may not accurately mirror the host immune response and disease progression observed in ONNV patients (29). A wild-type C57BL/6J model for ONNV (SG650 strain) was previously attempted (29). While the route of ONNV inoculation and the age of the mice were comparable to the 6WO model in this study, the lack of joint swelling and detectable viremia is intriguing. This could be due to the 1000-fold less virus inoculated into the footpad, as the adopted dose (10^3^ PFU) mimic virus inoculation load during a typical mosquito bite (29). However, the lack of ONNV-induced pathology limits it as a model for validating prophylactic and therapeutic strategies. Thus, an increase in ONNV dose is necessary to establish an *in vivo* model that can recapitulate virus-induced disease observed in patients. Nevertheless, pathogenicity of IMTSSA/5163 and SG650 strains are likely similar, given their close phylogenetic relationship (11). The risk of global transmission, coupled with the lack of immune-competent models to study ONNV infection, highlights the need of a suitable pre-clinical *in vivo* model to explore therapeutic and prophylactic strategies.

Here, an immune-competent *in vivo* young adult mouse model for ONNV infection is reported. When infected with ONNV, animals recapitulated the self-limiting symptom profiles seen in patients, i.e., the viremia which clears by 8 dpi and arthralgia peaking at 6 dpi (Figures 1A,B) (1, 5, 30). ONNV joint pathology was characterized by marked muscle degeneration and necrosis, mild synovitis, and tenosynovitis around the tendon capsule of the infected joint. However, joint swelling was not associated with edema (Figure 1C). This mirrors what is observed in virus-infected patients where edema is also not observed (30). This unique observation sets ONNV apart from old-world arthritogenic alphaviruses like CHIKV, Mayaro virus, and Ross River virus, where edema is frequently or occasionally observed (31, 32). Therefore, this immune-competent mouse model can be appropriately adopted for understanding ONNV disease pathogenesis and the discovery of therapeutic strategies.

High-dimensional UMAP and flow cytometry analysis of leukocyte populations highlighted the increase in proportion and quantity of CD4+ T cells in ONNV-infected joints. Depletion of CD4+ T cells drastically reduced joint footpad swelling, immune cell infiltration and muscle degeneration and necrosis at 6 dpi (Figures 3A,C,D), with no impact on viremia clearance (Figure 3B). Similar pathogenic effects of virus-specific CD4+ T cells were previously reported for other alphaviruses, including CHIKV (16, 21, 26, 28), and Sindbis virus (SINV) (27), suggesting that targeting CD4+ T cells is a viable treatment option for patients suffering of ONNV-induced arthralgia. To confirm this possibility, we investigated if FTY720, an FDA-approved T cell infiltration suppressive drug could be used to reduce ONNV induced joint swelling. Administration of FTY720 into ONNV-infected joints reduced the joint swelling during the peak of inflammation at 6 dpi (Figure 5). We previously showed that FTY720 could effectively reduce CHIKV-induced arthralgia (28), suggesting that this strategy could be applicable to a large spectrum of arthritogenic alphaviruses. Successful treatment of alphavirus disease using CTLA4-Ig, an anti-rheumatic drug has also been reported to suppress CD4+ T cell activation in the CHIKV-infected joint (26) and it would be interesting to assess its potency during ONNV infection. In general, the anti-rheumatic effects of such FDA-approved drugs, together with their wide usage against multiple sclerosis and rheumatoid arthritis, respectively, highlights the interesting possibility of repositioning immune-modulating drugs targeting the pathogenic CD4+ T cell response for these virus-induced joint and muscular pathologies. However, the use of such drugs is only effective if the CD4+ T cells are upregulated and major contributors in alphavirus-induced disease in patients. Thus, we propose the use of [^18^F]FB-IL-2 PET imaging (20), a non-invasive screening method that can be used to characterize activated CD4+ T cell profile and their pathological role in alphavirus infected patients. This will allow accurate prescription of immune-modulatory drugs against the identified pathogenic mediator. Similar repositioning of drugs that target viral protein synthesis, increase viral genome mutational load or decrease virus infectivity has previously been performed successfully for different viral pathogens, such as dengue virus (33), human immunodeficiency virus (34) and zika virus (35).

In light of the underestimated ONNV epidemics that is still currently on-going and the increase potential of world-wide outbreaks, this immune-competent mouse model will be an invaluable tool for *in vivo* characterization of the mechanisms between the immune driven pathogenesis as well as assessment of FDA-approved drugs repurposing efficiency.

## Data Availability Statement

The datasets generated for this study are available on request to the corresponding author.

## Ethics Statement

The animal study was reviewed and approved by Institutional Animal Care and Use Committee (IACUC 181353) of A^*^STAR Agri-Food and Veterinary Authority (AVA) National Advisory Committee for Laboratory Animal Research of Singapore (NACLAR).

## Author Contributions

LN and LR supervised the study. Y-HC and T-HT conceived and designed the experiments. Y-HC, T-HT, AT-R, RC, and F-ML performed the experiments. RR performed blind histopathological scoring of the virus infected joints. Y-HC, T-HT, AT-R, RR, F-ML, and GC performed data analysis. SK, FY, and ER were responsible for production of [^18^F]FB-IL-2. SH, BR, PC, and JG were responsible for PET imaging and analysis. Y-HC, GC, LR, and LN wrote the manuscript. All authors viewed and approved the manuscript.

## Conflict of Interest

The authors declare that the research was conducted in the absence of any commercial or financial relationships that could be construed as a potential conflict of interest.

## Supplementary Material

Supplementary Video 1 Longitudinal profiling of [^18^F]FB-IL-2 uptake in FTY720-treated mice.
