# Supplementary figures and images for: Longitudinal [18F]FB-IL-2 PET Imaging to Assess the Immunopathogenicity of O'nyong-nyong Virus Infection
Source: Front Immunol. 2020 May 12;11:894. doi: 10.3389/fimmu.2020.00894 (PMC7235449; doi:10.3389/fimmu.2020.00894)

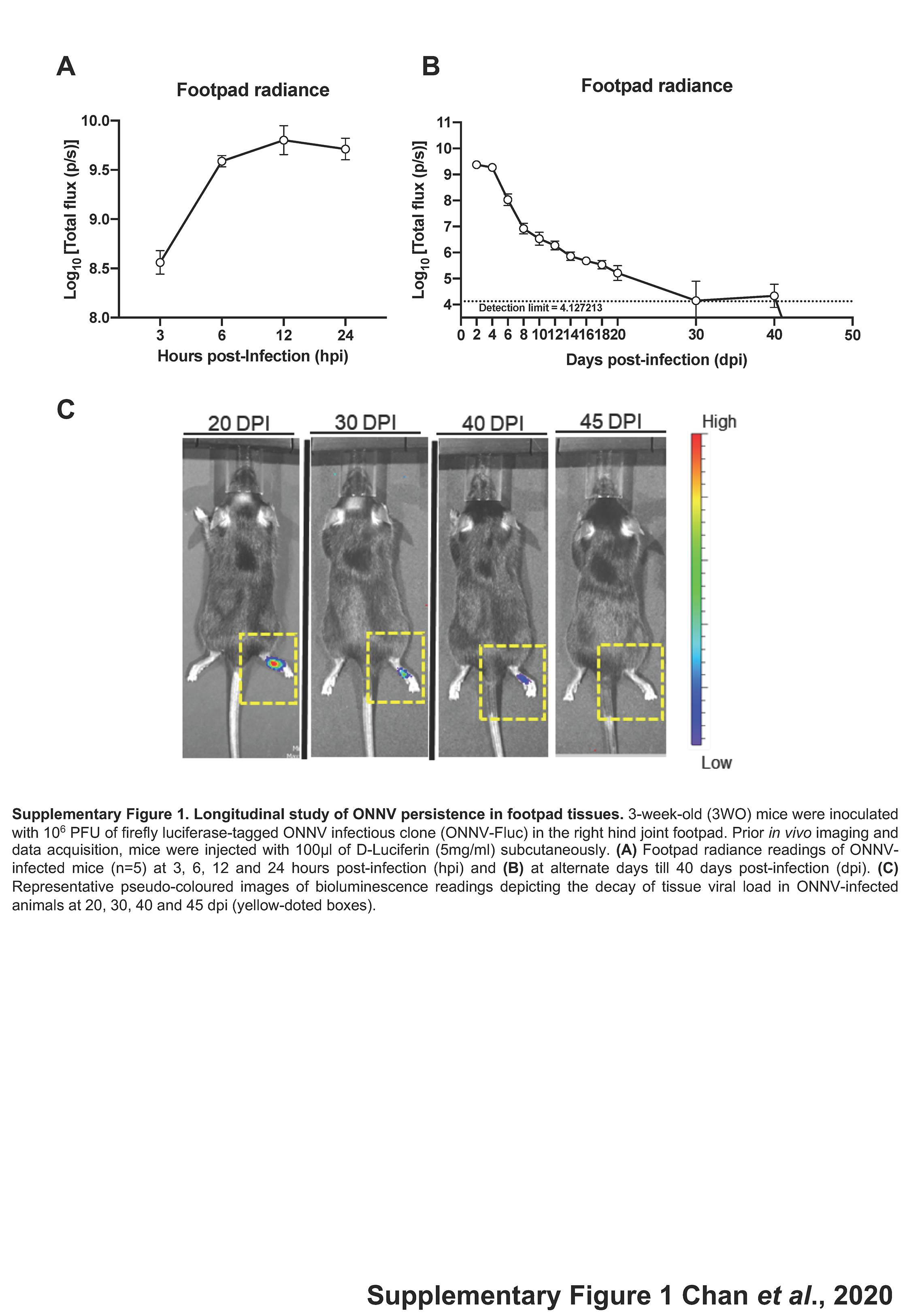

Supplement: Supplementary file 2 [file Image_1.TIFF]

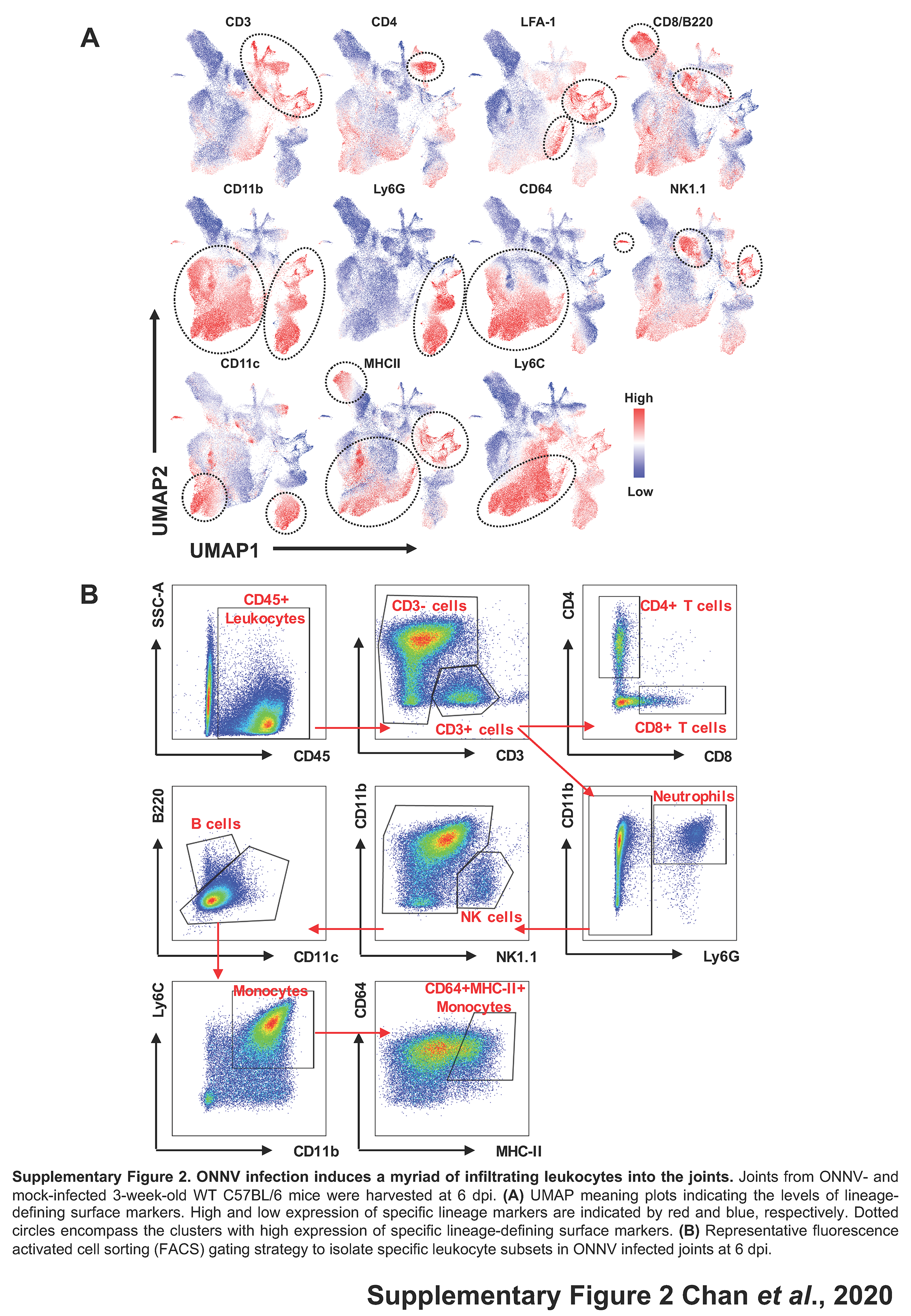

Supplement: Supplementary file 3 [file Image_2.TIFF]
